# Modifying mosquitoes to suppress disease transmission: Is the long wait over?

**DOI:** 10.1093/genetics/iyac072

**Published:** 2022-06-02

**Authors:** Jeffrey R Powell

**Affiliations:** Yale University, New Haven, CT 06511, USA

**Keywords:** vector genetics, vector-borne diseases, genetic control, transgenic mosquitoes, *Aedes aegypti*, GM mosquitoes, Anopheles, Wolbachia, selective breeding

## Abstract

For more than 50 years it has been a dream of medical entomologists and public health workers to control diseases like malaria and dengue fever by modifying, through genetics and other methods, the arthropods that transmit them to humans. A brief synopsis of the history of these efforts as applied to mosquitoes is presented; none proved to be effective in reducing disease prevalence. Only in the last few years have novel approaches been developed or proposed that indicate the long wait may be over. Three recent developments are particularly promising: CRISPR-Cas9 driven genetic modification, shifting naturally occurring allele frequencies, and microbe-based modifications. The last is the furthest along in implementation. Dengue fever incidence has been reduced between 40% and 96% in 4 different regions of the world where Wolbachia-infected *Aedes aegypti* have been established in the field. It is not yet clear how sustainable such control programs will prove to be, but there is good reason for optimism. In light of this, the time is ripe for reinvigorated research on vectors, especially genetics. Vector-borne diseases primarily affect under-developed countries and thus have not received the attention they deserve from wealthier countries with well-developed and funded biomedical research establishments.

## Introduction

In the summer of 1968, a workshop was held at the University of Notre Dame jointly sponsored by the University and the World Health Organization. The title of the course was “Seminar in Vector Genetics” and focused on genetics of insects that transmit pathogens. At the time, genetics as a subfield of medical entomology was in its infancy, but great hope and excitement abounded concerning the potential use of genetics in controlling insect vectors. The WHO sponsored 25 medical entomologists from around the world to attend this 6-week course. A second wave of excitement occurred between 1985 and 2000 when the MacArthur Foundation used its resources to re-invigorate the field and to attract new researchers, largely molecular biologists, to vector genetics (Beaty *et al.* 2009). While great advances in understanding vectors have been made, attempts to harness these advances to effectively limit or control vector-borne diseases have been disappointing. In fact, it is fair to conclude that, with one notable exception (discussed below), *there has been no successful and sustained vector modification program that has reduced incidence of human diseases.*

Here, I briefly review efforts made over the last 50+ years to use mosquito modification to control vector-borne diseases. I then point out signs of hope that the long dreamed-of approach may finally become a reality. To limit the presentation, I will focus on mosquitoes although many of the principles discussed apply more generally to vectors of diseases, especially arthropods.

## Brief synopsis of 50 years of frustration

### Suppresion vs replacement

It is important to distinguish 2 categories of control of vector-borne diseases targeting the vector: decrease the number of vectors (population suppression) or genetically change the vectors so they have reduced or no capacity to transmit pathogens (population replacement). Initially, almost all efforts in genetic control were aimed at reducing numbers. Indeed, a WHO working group (1964) defined genetic control as “….the use of any condition or treatment that can *reduce the reproductive potential* of noxious forms by altering or replacing the hereditary material.” (Emphasis added.)

The first, largely successful, attempts to reduce numbers of harmful insects using a genetic approach were for agricultural pests, the screw worm and then fruit flies (reviewed in Scott *et al.* 2017). At the time, the 1960s, X-rays were known to sterilize insects, with male spermatogenesis being more vulnerable than oogenesis. Fairly low dosages of radiation could be used leaving the sterilized males not too debilitated to compete for females in the field. Rearing in the lab and release of massive numbers of sterile males became known as the sterile insect technique, SIT.

### SIT for vectors

With the success of SIT for agricultural pest insects, mosquitoes were quickly targeted for similar work. Various methods were used to obtain sterile male mosquitoes including irradiation, chemosterilization, hybrid sterility, and chromosomal rearrangements (e.g. translocations). Males have always been the choice for releases as only females take blood meals so releasing males does not increase disease transmission or nuisance level. Benedict and Robinson (2003) reviewed 28 ensuing mosquito SIT programs targeting 10 mosquito species. Most of these were done in the 1960s and 70 s with the most recent being 1991, 31 years ago. The fact that the technique has been largely abandoned, despite a considerable rise in many mosquito-borne diseases, is stark testimony to the ineffectiveness or impracticability of this technology for controlling mosquito-borne diseases. SIT was effectively abandoned for mosquitoes, although still effectively used for agricultural pests.

A modified form of mosquito SIT was revived in the 21st century with the development of novel methods based on transgenic manipulations. A commercial company in England (Oxitec, Ltd.) transgenically engineered a strain of *Aedes aegypti* that could produce males homozygous for a dominant lethal, a method they dubbed release of insects with dominant lethals, RIDL (Alphey and Andreasen 2002). While not technically sterile, because (most) offspring sired by the released modified males died before the adult stage, RIDL is effectively the same as SIT. The results of RIDL in a number of localities in Brazil and Asia have been reasonably successful with temporary reduction in small target populations (<200 houses) up to about 85% although, averaged over time, reduction is closer to 50–60% (Carvalho *et al.* 2015; Garziera *et al.* 2017). One undesired finding was that genetic material can be transferred from the transgenic release strain into the target population (Evans *et al.* 2019) heightening concerns from groups opposed to use of transgenic GMOs. There is no evidence that RIDL has had an effect on mosquito-borne diseases nor has it been shown to be sustainable. (A microbe-based SIT program discussed later does show promise.)

### Producing benign vectors

Genetically manipulating or modifying organisms to the benefit of humans has a long history especially in agriculture. The ethics and potential risks of such work has been called into question in recent decades under the general rubric of GMOs, genetically modified organisms. However, many such concerns arise due to conflation of meanings of the term “genetically modified.” Through plant and animal selective breeding for centuries, virtually every commercial crop or food animal used by humans today has been genetically modified as have all our pet dogs and cats. The genetic changes induced over generations are due to *changing the frequencies of genes (alleles) already present in the species* or if carried out long enough, new spontaneous mutations arising in the selected strain(s). Most anti-GMO activists would stop short of advocating elimination of all Holstein cows or border collies. The concern over GMOs has arisen largely due to the more recent technique of *transgenesis:* the introduction of exogenously derived DNA not normally a part of a species genome. Virtually all recent attempts at genetic modification to control of vectors have involved transgenesis.

Genetically manipulating vectors so they have reduced or no capacity to transmit pathogens (said to be refractory) is a highly attractive approach to control vector-borne diseases. Many of the limitations of population suppression may be avoided. Most importantly, replacement control programs may be more stable and not require continuous application: once a target population has been replaced by desired genotypes further releases may not be required, or only intermittently required. Stability depends mostly on the fitness of refractory genotypes relative to susceptible in the field. This has 2 facets: (a) the relative fitness of the 2 types even in the absence of pathogens and (b) the possible fitness cost of being infected. For the first issue, as noted below, to date, most genetic manipulations producing refractory mosquitoes result in lowered fitness. Concerning (b), for most combinations of mosquito vector and pathogen, there is no obvious selective advantage or disadvantage to the vector to be refractory or susceptible. Infection with a relatively large protozoan like Plasmodium (malaria) would be the most likely to reduce female fitness; a review of literature of lab studies addressing this concluded that there is little or no evidence of reduced fitness due to infection (Ferguson and Read 2002). Except for equine encephalomyelitis virus (Weaver *et al.* 1988; Scott and Lorenz 1998), there is no evidence that arboviral infections reduce female mosquito fitness. If there is a cost to infection, it would be favorable to establishing and retaining refractory genotypes.

A second desirable attribute of quality control is that the targeted mosquito species remains in its ecological niche inhibiting immigration of an alternate mosquito vector and negating any ecological disturbances caused by removal, or near removal, of a species.

Using transgenesis of vectors to control disease requires 2 major steps: (1) isolate DNA sequences that, when placed in a vector, renders them partially or totally refractory to transmitting a pathogen and (2) have carriers of this transgenic construct replace (or largely replace) the existing susceptible genotypes in a field population. The first step is done by laboratory manipulations such as transformation, experimentally inserting a fragment of DNA into the genome of a living organism. The second step has largely been attempted by attaching this gene construct to a “drive” mechanism, a way to spread the new refractory genotypes into a field population.

### Transposable elements

Technologies to purposely and specifically change DNA in eukaryotes (transformation), especially in the germ line so changes are inherited, only became possible with the recognition of transposable elements (TEs) in the 1980s. These are small segments of virtually all eukaryotic genomes that have the ability to move around genomes, sometimes called “jumping genes.” If one cloned such an element and attached to it a gene or DNA sequence that had a desirable property (e.g. caused a vector to be incapable of transmitting a pathogen), in theory one could make vectors harmless or at least less harmful. While TEs are found as a normal part of the genome of vectors, most work on vectors transformation has used TEs and attached DNA from other species making the resulting animals transgenics.

What was particularly attractive about TEs is that some are capable of driving themselves through a population by replicating and inserting in other places in a genome. Examples of the P- and I-elements in *Drosophila melanogaster* were the seductive model. These TEs were unknown in this species before 1925 and by about 1980 almost all *D. melanogaster* collected in nature had these elements, due to natural spread with no human intervention (Figure 1 in Kidwell 1983). Could similar TEs be found in vectors or inserted in vectors and spread? It is not a coincidence that the MacArthur Foundation began their support of vector genetics at about this time.

Over about 15 years, 1985–2000, methodologies to transform many of the major arthropod vector of human diseases using TEs were developed. Dong *et al.* (2022) list 24 such studies using TEs to insert genes into Anopheles mosquitoes that suppress or eliminate malaria parasite development; all had fitness costs compared to nontransformed mosquitoes. While some of these were tested for their ability to drive through populations in the laboratory, no successful TE-genetic modification of field populations of vectors has been reported. Similar to SIT, this line of research has been largely abandoned.

## Hope

### CRISPR

Around 2012, a radically new technology to genetically manipulate virtually any genome was developing, CRISPR-Cas9. The great advantage of this method is that it is much more efficient than using TEs, can be used to specifically target places in a genome to insert exogenous DNA, and by attaching an appropriate enzyme gene, can spread itself by transforming homologous chromosomal segments. In a heterozygote for the presence of the CRISPR construct, when pairing in meiosis, the homologous region is filled in with the CRISPR and its attached DNA. Heterozygotes become homozygotes, a very efficient way to increase copy numbers in a population especially when initial frequencies are low. These properties are described in greater detail in Esvelt *et al.* (2014).

Considerable research is ongoing on using CRISPR to genetically modify vectors. Much of this work has focused on anopheline malaria vectors. One reason why this is a particularly attractive system for CRISPR control is that several single genes or regions of DNA have been identified that either make female refractory to Plasmodium transmission or cause lethality or sterility (Nolan 2021). The sterility-induced method has been shown to cause laboratory populations to go extinct (Kyrou *et al.* 2018) and a CRISPR-modified Plasmodium resistant strain has been shown to remain stably inherited through multiple back-cross matings with wild-type mosquitoes (Gantz *et al.* 2015). The only release of any genetically modified anopheline mosquitoes into the field (Burkina Faso) was a SIT attempt that indicated reduced fitness of CRISPR-modified sterile males (Yao *et al.* 2022).

Problems arise in scaling up such systems from individuals to genetically variable natural populations as opposed to limited lab populations. These systems have recognition or target sites where the insertion specifically takes place. Even though insertion sites are usually chosen because they are unique in a strain developed for replacement, variation exists in natural populations, off-site sequences and mutation may destroy targets or produce new target sites. Theoretical work indicated resistance to CRISPR invasion is likely to evolve in populations (Unckless *et al.* 2017). Laboratory studies of Drosophila confirm rapid resistance evolution (Champer *et al.* 2017).

Research on methods to avoid evolution of resistance to CRISPR invasion is an active field and may eventually circumvent resistance (Noble *et al.* 2018; Garrood *et al.* 2020). The existence of a very large database of mosquito complete genome DNA sequences makes it possible to identify highly conserved potential target sequences. Schmidt *et al.* (2020) have found that 90% of all protein-coding genes across 3 species of mosquitoes have at least one conserved potential target site with variants within a species being less than 1% across more than a thousand genomes. Nolan (2021) suggested conserved sites *across* species would also be a strategy to identify particularly stable unique targets.

Resistance to CRISPR invasion may also be due to mutations in the target site, either single nucleotides or insertion/deletions. Using Drosophila as a model, methods to restore effectiveness of invasion by repairing the resistant alleles have been developed (Champer *et al.* 2020a; Oberhofer *et al.* 2019) and successfully used in lab populations (Champer *et al.* 2020b). Adolfi *et al.* (2020) successfully adapted this approach to use in Anopheles and showed it to work in lab populations.

Thus, there is good reason to believe the 2 major obstacles to employing CRISPR-type technologies to modify natural populations, multiple target sites, and destruction/creation of target sites by mutation, can be overcome (Carballar-Lejarazú *et al.* 2020). Employment of these methods in natural populations can be anticipated and are awaited with great hope.

### Genetic shifting

As noted above, there are 2 types of genetic modification, transgenesis and selective breeding. While the former has received considerable attention as a means of producing benign vectors, the latter has not. Powell and Tabachnick (2014) proposed that such a program would have many advantages over transgenic methods and dubbed the approach *genetic shifting*, the shifting of allele frequencies already present in a species. The proposal was to select for a strain of a vector that was incapable of transmitting a pathogen by several generations of selective breeding based on the fact that, for almost all vectors examined, there is considerable genetic variance within and between populations for ability to be infected and transmit human pathogens. Attempts to select strains of mosquitoes with reduced ability to transmit pathogens have been successful, e.g. *A. aegypti* and dengue (Bennett *et al.* 2005) and *Anopheles gambiae* and malaria (Collins *et al.* 1986). Such selected strains could be released in large numbers to replace existing genotypes with higher capacity to transmit diseases.

No attempts to release such refractory strains have been attempted as it was not clear whether this could work especially because no “drive” was involved. What kind of numbers, over what period of time, would need to be released? Xia *et al.* (2019) modeled such a program assuming the variation in ability to transmit is a continuously distributed trait with multigenic underpinnings, i.e. a typical quantitative trait. Various parameter spaces were explored using realistic values based on *A.* *aegypti.* The results are remarkably encouraging. Modeling vector competence as a continuously distributed quantitative trait, a release of the selected refractory strain of 10% of the target population each generation for 20 generations, lowered the competence to transmit about 3 standard deviations. Assuming the genetic shifting has not caused a significant decrease in fitness, the lowered competence persists for many generations even after releases cease. Another variable explored was how different release strategies affect the rate of replacement. The most effective and rapid is when both males and females are released, with females having already received a blood meal before release. This reduces female threat to transmit as well as primes them to make a high contribution through egg laying. Modeling indicates any adverse effects of releasing prefed females is outweighed by number of disease cases avoided by speeding up the process (Xia *et al.* 2021).

A major advantage of this approach is it is applicable to any vector-borne disease when the vector can be reared in the lab and tested for ability to transmit. Decades of research on anophelines and malaria were a prelude to bringing that system to the point of using the CRISPR drive system that requires identification of an individual gene or DNA fragment that significantly reduces competence (Li *et al.* 2013). So far, such single functional units conferring refractoriness for other pathogens such as viruses have not been identified and may not exist, so CRISPR and related methods may not be applicable. Multigenic control of refractoriness/susceptibility is more likely and genetic shifting or related methods will be needed. Second, because the shifting method relies on naturally occurring genetic variation, no novel genotypes are released into the environment, circumventing public concerns.

### Microbiomes

Insects, including vectors of diseases, have long been known to harbor a plethora of microorganisms (microbiomes) as part of their normal existence. Some of the more interesting are ones that are inherited across host generations, often endosymbionts that live in egg cytoplasm (ooplasm). An alternative to modifying the genomes of vectors themselves is to use an inherited microbe that has desirable properties such as reducing pathogen transmission; the microbe itself may or may not be genetically modified.

One of the more widespread insect endosymbionts are bacteria in the genus *Wolbachia* which have been found in 52% of all arthropods examined (Weinert *et al.* 2015). In most cases, the infection and transmission are benign. However, A. Ralph Barr and associates demonstrated that Wolbachia infections can have surprising effects, in particular, being the causative agent of incompatibility in crosses between distant populations of the *Culex pipiens* complex of mosquitoes (Yen and Barr 1973), the causative bacteria being called *Wolbachia pipientis*. The term incompatibility is used to indicate that embryos are formed but die before full development. Martinez *et al.* (2021) have elucidated details of the genetics underpinning this phenomenon.

An unexpected property of *W. pipientis* is that when the mosquito vector *A.* *aegypti* (that does not naturally carry Wolbachia; Gloria-Soria *et al.* 2018) is infected in the lab with Wolbachia, it significantly reduces the ability of this otherwise highly efficient vector to transmit viruses such as dengue, yellow fever, and chikungunya (Moreira *et al.* 2008). If inserted into the ooplasm, it is maternally inherited in *A. aegypti.*

### Microbe replacement control

Several release programs to establish Wolbachia infections in natural population of *A. aegypti* have been undertaken. The public concern of release of transgenic organisms are largely moot in this case. The genomes of the released mosquitoes are not changed and the only difference is a bacteria already widely present in all environments.

Because females transmit the infection, one way to drive the infection into a population is to release many infected males into a population with even a minority of infected females. Females with the infection are fertile with both infected and noninfected males, while uninfected females are sterile with infected males, thus giving female infection a selective advantage. Extensive modeling has been done to determine the conditions under which Wolbachia can become virtually fixed in a population (Ross *et al.* 2019). These programs aimed at population replacement have been dubbed *CI+blocking, female releases*.

Wolbachia infections in *A. aegypti* have been established in a number of field sites. One convenient property of the system is that females infected with Wolbachia in the laboratory can be outcrossed to males from the target field population. The resulting strain used for releases is thus genetically nearly identical to the target population but 100% infected.

The oldest such program with demonstrated persistence (8 years now) has been in and around Cairns, Australia (Hoffmann *et al.* 2011; Ryan *et al.* 2019). Wolbachia infection in the mosquito populations remained nearly 100% over this period with a ∼96% reduction in dengue cases. In Yogyakarta, Indonesia, with mosquito Wolbachia infection rates near 100% over 2 years, a reduction in dengue of about 77% was observed (Utarini *et al.* 2021). Importantly, protection was against all 4 dengue serotypes present at this time. In Kuala Lampur, Malaysia, Wolbachia infected mosquitoes averaged about 80% but fluctuated considerably over the year of the study. Dengue reduction was about 40% (Nazni *et al.* 2019). In Niteroi, Brazil, despite the level of infection of *A. aegypti* with Wolbachia being only 40–80%, a reduction in dengue incidence was 69% (Pinto *et al.* 2021). In addition, chikungunya was reduced by 56%. This demonstrates that even incomplete mosquito Wolbachia infection can reduce disease transmission as well as be effective against the other major viral disease, chikungunya.

*These are the* *first demonstrations that any kind of modification of vectors decreased human disease incidence.*

### Microbe population suppression

Wolbachia has also been used to reduce population sizes. Like in *C.* *pipiens*, Wolbachia-infected *A. aegypti* males crossed with uninfected females produce no viable offspring. Using this to reduce populations is called *CI only, male releases* or IIT, incompatibility insect technology. Crawford *et al.* (2020) reared in mass a strain of *A. aegypti* infected with Wolbachia that when mated to wildtype females produce no offspring. Females with ooplasm with Wolbachia were backcrossed using males from the target population (Fresno, California), a very efficient way to insure the release strain is genetically nearly identical to the target population. These were released over a large area in and around Fresno with a 95.5% (95% CI, 93.6–96.9) reduction of females in the target area.

A similar release program was carried out in northern Queensland, Australia (Beebe *et al.*, 2021). This release program needed to be modified taking into consideration that the targeted populations had already been subjected to a Wolbachia release program designed to establish Wolbachia infections in the wild to reduce disease transmission (see above). A different Wolbachia strain was required that produces males incompatible with both Wolbachia-carrying and noninfected wildtype in the targeted populations. The program was largely successful with 80%+ reduction in adult population sizes. For the most isolated population subjected to the least immigration, the reduction carried over to the next season between which no releases were made.

Why are these SIT-type control programs successful when previous attempts largely failed? Male mosquitoes are not rendered sterile by debilitating irradiation, chemosterilization, or transgenic modification. They are simply incompatible with the majority of the target population females. Second, the released strains can be easily backcrossed to the target population(s) assuring that in addition to being robust, the released males are genetically nearly identical to males in the natural population. It is simpler to make release strains genetically nearly identical to the target populations with a cytoplasmic factor than for nuclear constructs: there is no need to assay each generation for those offspring carrying the construct. Third, an efficient and accurate automated system was developed to separate males from females allowing releases of only males with less effort. The inadvertent release of even a single infected female could seriously compromise this approach.

## Prospects and limitations

Vector-borne diseases comprise 3 (or more) tightly balanced species interaction systems that have complex evolutionary dynamics (Powell 2019). The failure of many modification control programs has been due to lack of appreciation of evolutionary dynamics and pressures. The successes are more attuned to these dynamics and hold out hope for ultimate success.

### Population suppression

Any attempt to reduce the number of vectors puts pressure on the vector to evolve resistance, e.g. insecticide resistance. In the case of SIT, the most obvious and often observed, is for females in the target population to evolve mating discrimination against males from the release strain. This has been documented in the agricultural pest SIT programs (McInnis *et al.* 1996; Scott *et al.* 2017) and likely occurred in at least 1 Oxitec RIDL mosquito release program (Powell 2018). While to date there is no evidence this has occurred in the IIT Wolbachia programs, continued releases may well lead to mating discrimination even if the release strain was initially genetically nearly identical to the target population. Long-term laboratory rearing is likely to cause some inbreeding and adaptations to the lab environment to genetically change the release strain. In the screw worm and fruit fly control programs spanning decades, decrease over time in effectiveness is observed; replacement of the release strain is routinely done to restore effectiveness. Similar restoration of effectiveness can be fairly easily overcome by outcrossing to the target population. As noted above, this is more easily achieved for a cytoplasmic factor than for a nuclear gene construct.

A second limitation of any control program aimed at reducing vector population sizes is they must be continuously applied. Ceasing control (for economic or logistic reasons) allows vector populations to rebound quickly to precontrol levels. Finally, reduction or elimination of 1 vector species in a locality may open the niche for invasion by another potential vector species.

### Population replacement

In the case of replacing competent vectors with refractory ones, the evolutionary pressure shifts to the pathogen to evolve ways to regain effectiveness of transmission to hosts, i.e. reproduce. This will be the case regardless of whether the genetic modifications are transgenic, based on natural variation (genetic shifting), or microbe-induced (e.g. Wolbachia). This is analogous to pathogens evolving resistance to chemotherapy such as Plasmodium to quinine and artemisinin. Arboviruses, in particular, have a high capacity for rapid evolution due to large population sizes and high mutation rates. There is no reason to think, for example, that the dengue virus cannot evolve to regain transmission in Wolbachia-infected *A. aegypti.*

There may also be selection on female mosquitoes to evolve a response to Wolbachia infection. Ford *et al.* (2019) demonstrated the presence of genetic variance in *A. aegypti* for level of Wolbachia-induced dengue blocking. Importantly, mosquitoes selected to have reduced blocking also had reduced fitness implying such changes in the effect of Wolbachia infections is unlikely to evolve naturally. Bull and Turelli (2013) address in detail expected evolutionary pressures associated with the Wolbachia approach.

### Contingencies

Given that the long-term use of almost any kind of control strategy will eventually breakdown due to evolutionary pressures, it is wise to have in place contingencies. This is similar to insecticide resistance or antibiotic resistance: changing the chemical can restore effectiveness. The ease with which a disease control program based on genetics or microbes can be modified to circumvent resistance depends greatly on the method. Using a highly modified transgenic strain may require years of laboratory work to develop alternatives, whereas the Wolbachia-based methods may be more flexible. Already it was demonstrated that it could be modified when the target population is already infected with Wolbachia, compare Crawford *et al.* (2020) and Beebe *et al.* (2021). Discrimination by females against mating with released males can be quickly remedied by outcrossing the release strain to males of the target population. Depending on how quickly strains can be selected in the lab to be refractory, the genetic shifting approach may be similarly flexible.

## Epilogue

Are we truly at or near the end of the saga to harness vector modifications to control the overwhelming disease burden imposed by mosquitoes and other vectors? Hardly, it is too soon to celebrate or to curtail investigation of other methods. It will probably take another 5 to 10 years to see how effective and long-lasting the Wolbachia-based control measures are. Will the kinds of evolutionary scenarios just discussed arise to thwart this progress? If so, can they be anticipated and have in place necessary adjustments to continue control?

Another important point is that *A.* *aegypti* is probably the easiest vector of human diseases to control using genetics largely because of its ease of rearing in captivity; this makes both laboratory manipulations and mass rearing for releases relatively easy. It is not surprising that this “low-hanging fruit” is the first success. Can bacterial infections be a model for other mosquitoes such as anophelines that transmit malaria? And in addition to mosquitoes, other insects transmit human diseases such as tsetse flies in Africa and triatomid bugs (Hemiptera) in South America transmitting trypanosomiasis. The genetic shifting approach is more generalizable and applicable to any vector that can be reared and infected in the laboratory.

Finally, it is important to address the question of whether all the work, careers, and millions of dollars devoted to genetic control of vectors have been largely wasted if so many approaches came to naught. While the ultimate goal of reducing disease by controlling vectors has nominally been the *raison d’etre* for these decades of effort, along the way 2 important things have happened. First, the number and diversity of researchers working on vectors has exploded. In 1968, virtually all vector biology was done by researchers trained as classical entomologists. Today, almost every stripe of researcher can be found actively working on vectors, from molecular biologists to ecologists to computer scientists.

This has led to the second major outcome, a true blossoming of our understanding of vectors. The entire field of insect innate immunity has developed largely stimulated by interest in how insects fight off pathogen infections, the infections that harm themselves and those that ultimately harm humans. Molecular biology of insect vectors was practically nonexistent 60 years and it is only in the last 35 years that the field became well-established with a vast literature. Vector genetics has also grown along with the entirely new field of vector genomics. Today, literally thousands of genomes of vectors have been sequenced and it will be a challenge for some years to come to mine all the information that now resides in these enormous databases.

One cannot help but be reminded of the “war on cancer” begun 60 years ago. Cancer, unfortunately, is still with us, but much of modern day molecular, cell, and developmental biology was a result of this “war.” Much of what we know about vectors today is owed to efforts undertaken, at least nominally, to develop vector modification to reduce disease transmission.
